# Ultra-processed food intake in relation to anthropometrics and biomarkers of cardiometabolic health among Swedish children: a cross-sectional study within the GraviD-Child cohort

**DOI:** 10.1186/s40795-026-01337-w

**Published:** 2026-04-28

**Authors:** Linnea Bärebring, Anna Amberntsson, Mathilda Forsby, Therese Karlsson, Frida Dangardt, Anna Winkvist, Hanna Augustin

**Affiliations:** 1 Department of Internal Medicine and Clinical Nutrition, Institute of Medicine, Sahlgrenska Academy, University of Gothenburg, Box 459, Gothenburg, 405 30 Sweden; 2 Department of Life Sciences, Division of Food and Nutrition Science, Chalmers University of Technology, Gothenburg, Sweden; 3 Department of Molecular and Clinical Medicine, Institute of Medicine, Sahlgrenska Academy, University of Gothenburg, Gothenburg, Sweden; 4 Pediatric Heart Center, The Queen Silvia Children’s Hospital, Sahlgrenska University Hospital, Gothenburg, Sweden

**Keywords:** Ultra-processed food, Childhood, Adiposity, Cardiometabolic health, Blood lipids

## Abstract

**Background:**

Evidence regarding health effects of ultra-processed food (UPF) in children is scarce and contradictory. Therefore, the aim was to assess the associations between UPF intake and anthropometric measurements and biomarkers of cardiometabolic health in 8-year-old children in Sweden.

**Methods:**

This cross-sectional analysis was conducted at the follow up of *N* = 167 8-year old children born within the Swedish pregnancy cohort study GraviD. The children’s diet was assessed through a questionnaire and nutritional data derived using the Swedish Food Agency Food composition database. UPF intake was defined according to the NOVA framework. Participants were categorized into sex-specific tertiles of UPF intake, based on their energy-adjusted total UPF intake expressed as servings per day. Intake of UPF from the following subgroups was also categorized: beverages, sweets/snacks, bread/cereal, and protein/meals. Anthropometric measurements included body mass index (BMI), waist circumference and fat mass measured by bioimpedance. Biomarkers of cardiometabolic health were measured in non-fasting venous blood samples and measurements of blood pressure. Total UPF intake and subgroups of UPF were assessed in relation to the outcomes using adjusted linear regression analysis.

**Results:**

Mean ± SD intake of UPF was 6.1 ± 2.1 servings/day and contributed to 56 ± 11% of the children’s total energy intake. Neither intake of total UPF nor subgroups of UPF was associated with anthropometric measurements. Participants in the highest tertile of total UPF intake had lower concentrations of low-density lipoprotein (LDL) cholesterol compared to those in the lowest tertile (-0.28 [-0.51– -0.04]) and lower ApoB/ApoA1 ratio (-0.07 [-0.12– -0.03]). Higher intake of UPF beverages was associated with higher C-reactive protein, while higher intake of UPF sweets/snacks was associated with higher High-Density Lipoprotein, higher ApoA1 and lower ApoB/ApoA1 ratio. Higher intake of UPF protein/meals was associated with lower total cholesterol, LDL and ApoB/ApoA1 ratio. Higher intake of UPF bread/cereal did not show any significant associations.

**Conclusion:**

In this cross-sectional study of Swedish 8-year-old children, UPF intake was not associated with anthropometric measures. Higher intake of total UPF and certain UPF subgroups, particularly protein/meals, was associated with a more favorable blood lipid profile. These findings underscore the heterogeneity of foods classified as UPF and suggest that their cardiometabolic associations in children may differ substantially by subgroup.

**Trial registration:**

The GraviD-Child study was registered at www.clinicaltrials.gov (NCT05228925, 2022–01-27).

## Background

Ultra-processed foods (UPF) are foods that have undergone extensive processing and contain added flavors, colors, and other additives to create a highly palatable product with long shelf-life [1]. Examples of UPF include soda drinks, sweets, breakfast cereals, many packaged snacks, ready meals, and fast-food items. The NOVA food classification system is commonly used to classify food according to the degree of processing. NOVA classifies foods and beverages into four groups, where group 4 is UPF, indicating the highest degree of processing [1]. While processing provides foods with long shelf-life, high palatability and possibly reduced food waste, concerns of negative health effects have been raised regarding the relatively high intake of UPF globally and in Sweden [2, 3]. UPF contributes to approximately 40–60% of energy consumed in Western countries among children, adolescents, and adults [4–6] and high UPF intake has been linked to excess energy intake [7] and risk of various non-communicable diseases in adult populations [8]. Due to these concerns, dietary guidelines that recommend limiting intake of UPF are emerging [9]. However, the recently revised Nordic Nutrition Recommendations do not include such a recommendation, as it was not viewed to add anything to the existing recommendations [10].

In adult populations, high UPF intake is associated with increased risk of obesity [11–13], all-cause mortality, cancer, cardiovascular disease and increased cardiometabolic risk factors [8]. Considerably less is known of associations between UPF intake in children in relation to cardiometabolic risk factors. While associations between high UPF intake and increased body mass index (BMI) and adiposity are fairly consistent between studies of children [14], associations with indicators of cardiometabolic health are less studied and less homogenous. Brazilian cross-sectional and prospective data on 7–10-year-old children show that high UPF intake is not associated with blood glucose, blood pressure or blood lipids [15–17]. In addition, Spanish cross-sectional data of children aged 3–6 years show that higher UPF intake is associated with lower high-density lipoprotein (HDL) cholesterol and higher fasting plasma glucose, but not with other cardiometabolic risk factors (e.g. other blood lipids, insulin resistance or blood pressure) [18]. Similarly, a prospective cohort study found no associations between UPF intake at 7 years of age and cardiometabolic risk factors at 10 years of age in Portuguese children [19]. In contrast, a prospective Brazilian study found that high UPF intake in preschool age was associated with higher total cholesterol and triglycerides in school age [20], and with increments in total cholesterol and low-density lipoprotein (LDL) cholesterol over time [21]. Lastly, cross-sectional analyses from the European multicentre study I.Family showed no associations between UPF intake and indicators of the metabolic syndrome in 6–9-year-old children [22]. Thus, data regarding associations between UPF intake and biomarkers of cardiometabolic health in childhood are both scarce and contradictory. The aim of this study was therefore to investigate the association between UPF intake and anthropometric measurements and biomarkers of cardiometabolic health (i.e. blood lipids, glycemic markers, inflammation and blood pressure) in 8-year-old children in Sweden.

## Methods

### Study design and setting

**Fig. 1:**
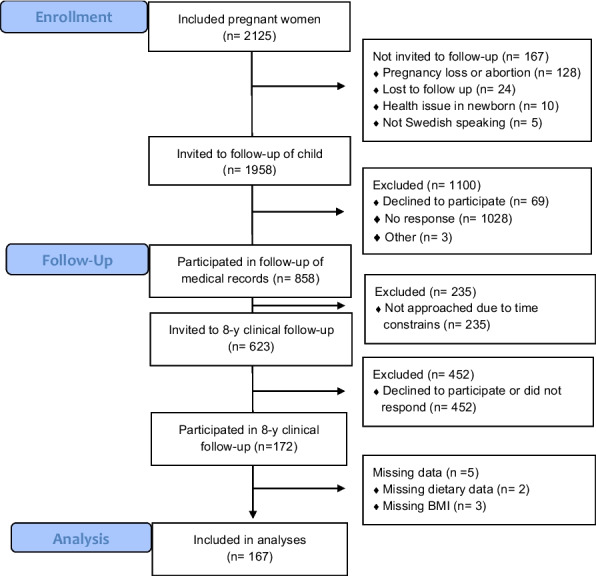
Flow chart illustrating the selection and recruitment of the children included in the study and current analyses

The GraviD study is a pregnancy cohort designed to investigate the role of maternal nutrition in relation to outcomes during pregnancy, delivery and childhood [23]. Women attending antenatal care during 2013–2014 in parts of South-west Sweden were eligible for inclusion. The cohort study included 2125 pregnant women. After delivery, medical records from antenatal and obstetrics care were collected and parents were asked for approval for the childcare health records to be retrieved, for follow-up of growth and development. Most of the parents who consented to giving access to their child’s childcare health records (*N* = 858) were also invited to participate with their child in the clinical follow-up (*N* = 623), the GraviD-Child study, when the children were 8 years old, in 2022–2023 (Fig. 1). All were not invited to the follow up due to time constraints. Parents were approached via mailed letters with study information and asked to participate in the clinical follow-up of the children at a study visit at the Queen Silvia Children’s Hospital in Gothenburg. Age-appropriate study information was given to both children and parents, aided by illustrations of what the examination would entail. Parents were also offered verbal information, and the aim was to obtain consent for their child to participate from both parents. The Regional Ethics Committee in Gothenburg and the Swedish Ethical Review Authority gave ethical approval for the GraviD study (897‐11, T439‐13, T085‐14, 2019‐05219) and the GraviD-Child study (2019‐05219, 2021–04194). All research was conducted according to the guidelines in the Declaration of Helsinki. The GraviD-Child study was registered at www.clinicaltrials.gov (NCT05228925).

### Data collection

Data collection of maternal sociodemographic factors was performed by questionnaires during pregnancy. Data regarding the pregnancy, including the child’s birth size and age at delivery, were collected through medical records. The clinical follow-up of the children at age 8 included questions on the children´s intake of food and measurements of anthropometry, cardiometabolic biomarkers and nutritional status. Physical activity was assessed using two questions regarding the duration and frequency of physical activity during leisure time [24] and expressed as hours per week. At the study follow-up visit, non-fasting measurements were conducted in a standardized sequence: height, weight, waist circumference, blood pressure, body composition, and blood sampling. Anthropometry was measured as height, weight to the nearest 0.1 kg and waist circumference to the nearest 0.1 cm and body composition by bioelectrical impedance analysis (Impedimed SFB7). Systolic and diastolic blood pressure was measured (Welch Allyn Connex® Vital Signs Monitor 6000 Series™). All measurements were repeated three times, and the results averaged. Blood was analyzed for total cholesterol, triglycerides, HDL cholesterol, LDL cholesterol (all using photometry) and lipoproteins Apolipoprotein A1 (ApoA1) and Apolipoprotein B (ApoB), (both using immunoturbidimetry), C-reactive protein (CRP, using immunoturbidimetry), plasma glucose, glycated hemoglobin A1c (HbA1c) (both using enzymatic photometry) and insulin (by immunochemistry) at the Sahlgrenska University Hospital. Childhood overweight (including obesity) was defined according to the International Obesity Task Force age-specific cut offs [25], where BMI > 25 kg/m^2^ at age 8 corresponds to 18.41 kg/m^2^ in boys and ≥ 18.28 kg/m^2^ in girls.

### Dietary assessment and UPF classification

Dietary intake was assessed using questions on food frequencies originally developed for 7 years old children in the Norwegian Mother, Father and Child Cohort Study (MoBa) [26] with minor changes to align with Swedish food culture. The questions were completed at home and brought to the study follow-up visit. The questionnaire included 50 food items and beverages and 6 frequencies of intake, ranging from never/seldom to ≥ 1 times/day for solid foods, and ≥ 4 cups/day for liquids. Nutritional intake was estimated, using half the standard portion sizes for adults and linkage to the Swedish Food Agency database (version 20.220.524). Protein intake was further divided into animal protein and plant protein, based on the predominant source (e.g. protein from bread, vegetables etc. was defined as plant protein and protein from meat, cheese, dairy, egg etc. was defined as animal protein).

UPF intake was classified by one nutritional researcher according to the NOVA system [1]. Assumptions made and food item classification are listed in Supplemental Table 1. Total UPF intake, defined as servings per day of NOVA category 4 food items, was energy-adjusted using the residual method. Further, UPF food items were classified into four subgroups of UPF: UPF bread/cereal, UPF protein/meals, UPF sweets/snacks and UPF beverages. Supplemental Table 2 lists which food items were included in each subgroup.

### Statistical analysis

Data are presented as mean ± SD or N (%). Total energy-adjusted UPF intake was expressed as servings/day, modelled as both sex-specific tertiles and continuous intake. Associations between UPF intake and anthropometric measurements and biomarkers of cardiometabolic health were assessed by linear regression analysis. Analyses of UPF subgroups were conducted using linear regression, with UPF intake modeled exclusively as a continuous variable to limit multiple comparisons. Logistic regression analysis was used to assess associations with overweight and obesity. All regression models were adjusted for potential confounders child sex, child physical activity, child gestational age at birth, birth weight as well as first trimester maternal age, BMI, and educational level. Analyses of UPF subgroups were further mutually adjusted for intake of the remaining UPF subgroups. Confounders were identified using directed acyclic graphs, based on previous literature. Model assumptions were assessed by inspecting distribution of residuals and heteroscedasticity in the associations, and CRP and insulin were transformed using the square root and natural logarithm, respectively, to comply with model assumptions. Tertiles of UPF intake in relation to lifestyle and sociodemographic data were tested using Kruskal Wallis test and Chi2 test for continuous and categorical variables, respectively. All statistical analyses were two sided and conducted in SPSS (version 29.0, IBM Corp, Armonk, New York, US). Statistical significance was accepted at *p* < 0.05.

## Results

A total of 167 mother–child pairs had complete dietary data and anthropometric measures of the children (Fig. 1). Biomarker data from blood samples were available for *n* = 139–141 children. The mothers were of mostly (88%) Northern European origin, were living with the other parent (97%), had full time employment (66%) and a university level education (77%). Among the children, mean breastfeeding duration was 10 ± 8 months and 46% were of female sex (Table 1).

**Table 1:** Background characteristics of women during pregnancy and their children

	All, *N* = 167
Maternal characteristics	
Maternal age in T1, years (mean ± SD)	32.8 ± 4.4
Maternal BMI in T1, kg/m^2^ (mean ± SD)	23.8 ± 3.5
Maternal university level education N (%)	128 (77)
Maternal employment status	
Full-time employment N (%)	109 (67)
Part-time employment N (%)	41 (25)
On parental leave N (%)	9 (6)
Unemployed N (%)	5 (3)
Co-parenting with partner N (%)	161 (97)
Maternal Northern European origin N (%)	147 (88)
Maternal overweight N (%)	53 (32)
Child charactaristics at birth and infancy	
Gestational age at birth, days (mean ± SD)	280 ± 12
Infant birth weight, grams (mean ± SD)	3544 ± 548
Infant sex, female N (%)	78 (46)
Breastfeeding duration, months (mean ± SD)	10 ± 7.6
Child characteristics at 8 years of age	
BMI (kg/m^2^)	16.1 ± 1.8
Waist circumference (cm)	56.1 ± 4.3
Fat mass (%)	21.1 ± 5.1
C-Reactive Protein (mg/L)	1.1 ± 3.3
Plasma glucose (mmol/L)	5.2 ± 0.6
Plasma insulin (mIE/L)	9.2 ± 6.8
HbA1c (mmol/mol)	31.8 ± 1.9
Total cholesterol (mmol/L)	3.7 ± 0.6
Triglycerides (mmol/L)	1.0 ± 0.6
HDL (mmol/L)	1.4 ± 0.3
LDL (mmol/L)	2.2 ± 0.6
ApoA1 (g/L)	1.5 ± 0.2
ApoB (g/L)	0.7 ± 0.2
ApoB/ApoA1	0.4 ± 0.1
Systolic blood pressure (mmHg)	102 ± 8
Diastolic blood pressure (mmHg)	64 ± 5

*BMI* Body mass index, *T1* Trimester 1

At the 8-year follow-up, 11% of the children had overweight. Total UPF intake was on average 6.1 ± 2.1 servings/day and contributed to 57 ± 11% of the children’s total reported energy intake (Table 2). The largest contributor to UPF intake, expressed as servings per day, was UPF bread/cereal (49%), followed by UPF protein/meals (16%), UPF sweets/snacks (15%) and UPF beverages (7%). Those with higher intake of UPF had significantly lower reported intakes of protein, animal protein, fat, saturated fatty acids (SFA), monounsaturated fatty acids (MUFA) and polyunsaturated fatty acids (PUFA). They also had a significantly higher reported intake of carbohydrates and plant protein (Table 2).

**Table 2:** Dietary intake and physical activity of the children at age 8 years, in relation to intake of UPF

	All Mean ± SD	Tertile 1^1^ Mean ± SD	Tertile 2^1^ Mean ± SD	Tertile 3^1^ Mean ± SD	P^3^
Child diet and physical activity					
Energy from UPF (%)	56.5 ± 10.5	47.6 ± 9.3	57.1 ± 7.9	64.6 ± 6.4	< 0.001
UPF intake (servings/day)^2^	6.1 ± 1.0	5.0 ± 0.6	6.0 ± 0.3	7.2 ± 0.5	< 0.001
Protein intake (E%)	17.0 ± 2.0	17.7 ± 1.7	16.7 ± 2.4	16.5 ± 1.7	< 0.001
Animal protein (E%)	9.4 ± 2.7	10.7 ± 2.6	9.0 ± 2.9	8.5 ± 2.3	< 0.001
Plant protein (E%)	7.6 ± 1.5	7.1 ± 1.7	7.6 ± 1.3	8.0 ± 1.5	0.002
Carbohydrate intake (E%)	54.4 ± 4.4	52.0 ± 4.0	55.3 ± 4.5	55.8 ± 3.8	< 0.001
Fiber intake (grams/day)	12.5 ± 4.3	11.3 ± 3.7	13.2 ± 4.7	12.9 ± 4.3	0.079
Fat intake (E%)	25.2 ± 3.8	27.1 ± 4.0	24.4 ± 3.3	24.2 ± 3.2	< 0.001
SFA intake (E%)	8.8 ± 1.8	9.7 ± 1.8	8.3 ± 1.5	8.4 ± 1.6	< 0.001
MUFA intake (E%)	9.5 ± 1.7	10.3 ± 1.9	9.3 ± 1.6	9.0 ± 1.4	< 0.001
PUFA intake (E%)	4.1 ± 0.9	4.4 ± 1.1	4.0 ± 0.8	3.8 ± 0.7	0.022
Physical activity (hours/week)	3.7 ± 2.6	4.1 ± 2.9	3.6 ± 2.6	3.3 ± 2.4	0.131
Maternal variables					
Maternal BMI (kg/m^2^)	23.8 ± 3.5	23.6 ± 3.7	24.1 ± 3.4	23.7 ± 3.3	0.638
Maternal overweight	53 (32%)	17 (31%)	20 (36%)	16 (29%)	0.710
Maternal university education	128 (77%)	45 (82%)	44 (79%)	39 (70%)	0.290
Maternal full-time employment	109 (67%)	36 (66%)	37 (66%)	36 (64%)	0.814

*UPF* Ultra-processed food, *E%* Energy percentage, *SFA* Saturated fatty acids, *MUFA* Monounsaturated fatty acids, *PUFA* Polyunsaturated fatty acids

^1^Cut offs for tertiles of UPF intake expressed as energy-adjusted servings/day; 1: < 5.48 for boys and < 5.64 for girls, 2: 5.48–6.50 for boys and 5.64–6.46 for girls 3: > 6.50 for boys and > 6.46 for girls

^2^Energy-adjusted using the residual method

^3^Continuous variables assessed by Kruskal Wallis test and categorical by chi square test

Total intake of UPF was not associated with anthropometry, CRP, measures of glycemia or blood pressure in either categorical or continuous models (Table 3). However, higher intake of total UPF was associated with lower LDL compared to lower UPF intake (B [95% CI]) (Tertile 3 vs Tertile 1: −0.275 [−0.513– −0.038]). Further, higher intake of total UPF was associated with lower ApoB/ApoA1 ratio in categorical (Tertile 3 vs Tertile 1: −0.073 [−0.119– −0.026] and Tertile 2 vs Tertile 1: −0.050 [−0.096– −0.004]) and continuous (−0.020 [−0.039– −0.001]) analyses.

**Table 3:** UPF intake expressed as energy adjusted servings/day in relation to anthropometry and cardiometabolic risk factors among the children at age 8 years

	**Tertile 1**	**Tertile 2**			**Tertile 3**			**Contiunous (servings/day)**		
		**β**	**P**	**95% CI**	**β**	**P**	**95% CI**	**β**	**P**	**95% CI**
BMI (kg/m^2^)	Ref	0.122	0.701	−0.505 – 0.749	0.213	0.505	−0.417 – 0.843	0.132	0.315	−0.127 – 0.392
Waist circumference (cm)	Ref	0.092	0.903	−1.392 – 1.575	0.524	0.484	−0.952 – 2.001	0.142	0.650	−0.474 – 0.758
Fat mass (%)	Ref	−1.227	0.162	−2.950 – 0.496	−0.200	0.820	−1.931 –1.531	−0.168	0.645	−0.886 – 0.550
C-Reactive Protein (sqrt) (mg/L)	Ref	0.023	0.848	−0.212 – 0.257	0.194	0.112	−0.046 – 0.433	0.057	0.247	−0.040 – 0.153
Plasma glucose (mmol/L)	Ref	0.004	0.974	−0.253 – 0.262	−0.047	0.721	−0.309 – 0.214	−0.092	0.081	−0.195 – 0.011
Plasma insulin LN (mIE/L)	Ref	0.004	0.981	−0.300 – 0.307	−0.035	0.822	−0.342 – 0.272	−0.058	0.349	−0.181 – 0.064
HbA1c (mmol/mol)	Ref	0.440	0.249	−0.312 – 1.191	0.487	0.209	−0.275 – 1.250	0.209	0.178	−0.096 – 0.514
Total cholesterol (mmol/L)	Ref	−0.155	0.208	−0.398 – 0.087	−0.186	0.139	−0.434 – 0.061	−0.041	0.417	−0.141 – 0.059
Triglycerides (mmol/L)	Ref	−0.091	0.436	−0.321 – 0.139	−0.081	0.497	−0.315 – 0.154	−0.009	0.848	−0.103 – 0.085
HDL (mmol/L)	Ref	0.021	0.704	−0.088 – 0.130	0.103	0.068	−0.008 – 0.214	0.034	0.134	−0.011 – 0.079
LDL (mmol/L)	Ref	−0.205	0.085	−0.438 – 0.028	−0.275	0.024	−0.513 – −0.038	−0.066	0.181	−0.162 – 0.031
ApoA1 (g/L)	Ref	0.02	0.695	−0.079 – 0.118	0.047	0.352	−0.053 – 0.147	0.012	0.542	−0.028 – 0.052
ApoB (g/L)	Ref	−0.06	0.101	−0.133 – 0.012	−0.06	0.111	−0.133 – 0.014	−0.015	0.323	−0.045 – 0.015
ApoB/ApoA1	Ref	−0.05	0.032	−0.096 – −0.004	−0.073	0.002	−0.119 – −0.026	−0.02	0.043	−0.039 – −0.001
SBP (mmHg)	Ref	0.304	0.841	−2.688 – 3.297	−0.665	0.664	−3.683 – 2.353	−0.31	0.628	−1.574 – 0.945
DBP (mmHg)	Ref	−0.749	0.388	−2.458 – 0.960	0.390	0.656	−1.334 – 2.113	0.000	0.999	−0.726 – 0.725

*UPF* Ultra-processed food, *BMI* Body mass index, *HbA1c* Glycated hemoglobin, *HDL* High density lipoprotein, *LDL* Low Density Lipoprotein, *ApoB* Apolipoprotein B, *ApoA* Apolipoprotein A, *sqrt* Transformed using the square root, *LN* Transformed using the natural logarithm, *SBP* Systolic blood pressure, *DBP* Diastolic blood pressure

Adjusted for child’s sex, child’s physical activity, child’s gestational age at birth, birth weight, maternal age, maternal BMI in the first trimester of pregnancy and maternal education

**Fig. 2:**
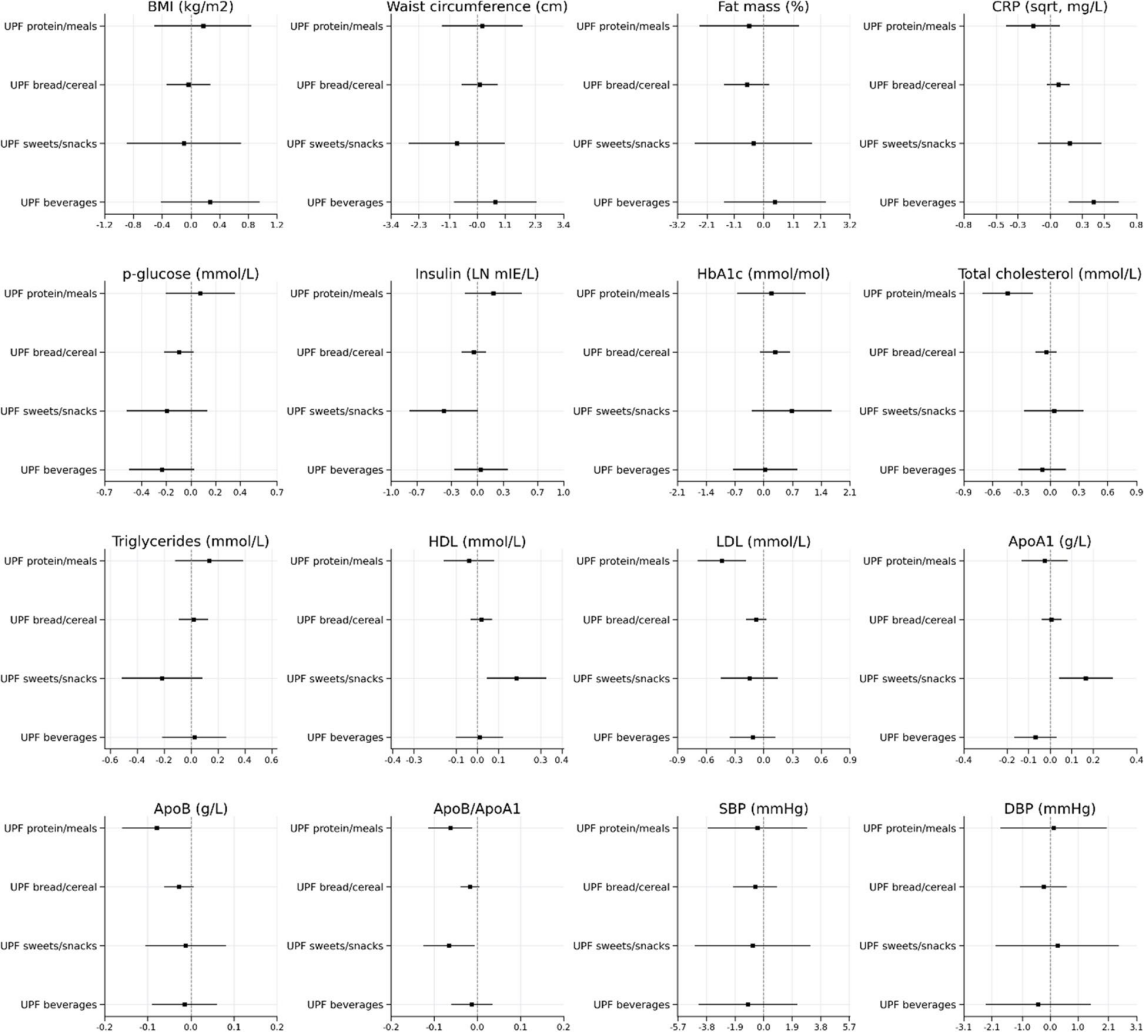
Beta and 95% confidence intervals from regression analyses of subtypes of ultra-processed food, expressed as energy adjusted servings/day, in relation to anthropometry and cardiometabolic risk factors among the children at age 8 years*.* All models are adjusted for energy intake (residual method), child sex, gestational age at birth, birth weight, physical activity, maternal education, maternal age and maternal BMI in the first trimester of pregnancy. All models are further mutually adjusted for intake of the other three subgroups of UPF. UPF; ultra-processed food, BMI; body mass index, HbA1c; glycated hemoglobin, HDL; high density lipoprotein, LDL; Low Density Lipoprotein, ApoB; Apolipoprotein B, ApoA; Apolipoprotein A, sqrt; transformed using the square root, LN; transformed using the natural logarithm, SBP; Systolic blood pressure, DBP; diastolic blood pressure

Analyses of intake of UPF subgroups showed no associations with anthropometric indicators. However, higher intake of UPF beverages was associated with higher CRP, while higher intake of UPF sweets/snacks was associated with higher HDL, higher ApoA1 and lower ApoB/ApoA1 ratio. Further, higher intake of UPF protein/meals was associated with lower total cholesterol, LDL and ApoB/ApoA1 ratio. Higher intake of UPF bread/cereal did not show any significant associations (Fig. 2). Neither total UPF intake nor intake of UPF subgroups was associated with overweight (Table 4).

**Table 4:** UPF intake expressed as energy-adjusted servings/day in relation to odds ratio of having overweight (including obesity) among the 8-year-old children

	**OR (95% CI)**	**P**
Total UPF intake (servings/day)^1^	1.106 (0.619–1.976)	0.733
UPF beverages (servings/day)^2^	1.523 (0.384–6.045)	0.550
UPF sweets/snacks (servings/day)^2^	0.936 (0.163–5.354)	0.940
UPF protein/meals (servings/day)^2^	0.908 (0.191–4.326)	0.904
UPF bread/cereal (servings/day)^2^	0.768 (0.393–1.499)	0.439

*UPF* Ultra-processed food

^1^Adjusted for child’s sex, child’s physical activity, child’s gestational age at birth, birth weight, maternal age, maternal BMI in the first trimester of pregnancy and maternal education

^2^Adjusted for child’s sex, child’s physical activity, child’s gestational age at birth, birth weight, maternal age, maternal BMI in the first trimester of pregnancy and maternal education, and for intake of the other three UPF subgroups

## Discussion

In this cross-sectional study of Swedish 8-year-old children, UPF intake was not associated with anthropometric measures or overweight. Higher intake of total UPF and certain UPF subgroups, particularly protein/meals, was associated with a more favorable blood lipid profile and lower intake of fat and SFA. These findings underscore the heterogeneity of foods classified as UPF and suggest that their cardiometabolic associations in children may differ by UPF subgroup and possibly also by the overall dietary context.

Previous research has shown disparate results regarding intake of UPF in association with anthropometry in children. A 2022 systematic review found that most of the included studies found no associations between UPF intake and obesity or adiposity in children, while some found positive or inverse associations [14]. We found no evidence of an association between UPF intake and anthropometric measures or overweight. However, the low prevalence of overweight in the current study may have limited the statistical power to detect such associations. Previous studies have also found disparate results regarding UPF intake in relation to cardiometabolic risk factors in children, and most have found no associations with glycemia, lipids or blood pressure [16, 17, 19, 27] or only with a subset of the outcomes [18]. However, Silva-Luis et al. found that among Brazilian children aged 7–10 years, those in the highest tertile of UPF intake had higher LDL cholesterol [28]. In addition, Rauber et al. [21] found that that higher UPF intake in children was associated with higher longitudinal trajectories of total cholesterol and LDL cholesterol. These results are contradictory to ours, as we found that LDL cholesterol was lower among children with high UPF intake. This may be due to the fact that, in the current study, higher UPF intake was associated with lower intake of SFA. Dietary intake is known to influence LDL cholesterol concentrations already in childhood [29] most likely through modifications in dietary fat quality, a mechanism that is well-documented in adult populations [30]. Since dietary fat quality and other nutritional aspects are not considered in the definition or classification of UPF according to NOVA, disparity in previous research is perhaps not unexpected. Major sources of UPF could differ in previous studies explaining, at least in part, disparities between different studies and geographical regions. Food choices are influenced by the overall food culture and context [31] which is important to consider when comparing results of UPF intake and health outcomes in different populations. Total UPF intake in the current study corresponded to approximately 50% of total energy intake which aligns with previous studies in children from countries such as Brazil [16] and New Zealand [32]. The major subtypes of UPF could however be drastically different between countries. Moreover, the nutritional quality of UPF subtypes may vary across countries, influenced by the specific food items most commonly consumed, which may in turn be shaped by regional dietary patterns and cultural food practices.

### Strengths and limitations

This study contributes to the limited evidence basis regarding associations between UPF intake and anthropometry and biomarkers of cardiometabolic health in children but has both strengths and limitations. Strengths of the study are the prospectively collected data regarding maternal and pregnancy factors, and a population based original cohort that enabled analysis of selection bias in the current follow up of the children. Limitations include the cross-sectional study design of the current analyses and the use of a short questionnaire for dietary assessment that has not been validated for nutrient intake nor intake of UPF and a single researcher classifying foods according to NOVA. The cross-sectional study design limits the ability to attribute observed associations to the exposure, as reverse causation cannot be ruled out. The lack of validation of the dietary questionnaire may have introduced measurement error in the assessment of dietary intake, potentially leading to misclassification of UPF consumption and an attenuation of true associations. The presented intake of nutrients should also be interpreted with some caution, with this in mind. Having a single researcher classify all food items according to the NOVA framework may have introduced classification bias. Additionally, all data on dietary intake and several covariates were self-reported which may have implications for their accuracy. Blood samples were collected in the non-fasting state which may limit precision in these analyses. We also lack comprehensive socioeconomic data, as maternal education level and employment status were the only available indicators, which may have resulted in residual confounding. Further, the study sample is not representative of the entire population, as evidenced by the low prevalence of overweight and obesity among the children (11% compared to the national average of 23% among 6–9 years old children [33]). In addition, maternal education level is also quite high, as almost 80% had a university level education, which may limit the external validity of the findings. In addition, the sample size is not substantial from a statistical standpoint, but relatively large within the context of clinical examinations of children. Nonetheless, it is possible that power to detect some significant associations is insufficient, which increases risk of type 2 error. Lastly, we performed several statistical tests, and the risk of type 1 error should be considered when interpreting the results. UPF intakes were in the analyses expressed as both categorial and continuous intake in order to enable comparisons to other studies and future meta-analyses. However, this further contributed to the number of statistical tests.

The current study adds to the growing body of evidence that shows that not all UPF subtypes are associated with worse health outcomes [34, 35]. Studies show that UPF bread and/or cereal are not detrimentally associated with health, and whole grain items reduce risk of ill health [36, 37]. Additionally, since both total UPF intake and intake of UPF protein/meals were associated with a more favorable lipid profile in the current study, the relevance of the UPF concept from a public health perspective can be questioned.

## Conclusion

In this cross-sectional study of Swedish 8-year-old children, UPF intake was not associated with anthropometric measures. Higher intake of total UPF and certain UPF subgroups, particularly protein/meals, was associated with a more favorable blood lipid profile. These findings underscore the heterogeneity of foods classified as UPF and suggest that their cardiometabolic associations in children may differ substantially by subgroup.

## Data Availability

Due to the European General Data Protection Regulation (GDPR) and that participants did not consent to their data being stored in public repositories, data cannot be freely shared. The datasets used analyzed in the current study are available from the corresponding author on reasonable request.

## Abbreviations

BMI: Body mass index
CRP: C-reactive protein
HDL: High-density lipoprotein
LDL: Low-density lipoprotein
MUFA: Monounsaturated fatty acids
PUFA: Polyunsaturated fatty acids
SFA: Saturated fatty acids
UPF: Ultra-processed foods
